# Basic parametric analysis for a multi-state model in hospital epidemiology

**DOI:** 10.1186/s12874-017-0379-4

**Published:** 2017-07-20

**Authors:** Maja von Cube, Martin Schumacher, Martin Wolkewitz

**Affiliations:** 1 Institute for Medical Biometry and Statistics, Faculty of Medicine and Medical Center - University of Freiburg, Stefan-Meier Str. 26, Freiburg, 79104 Germany; 2 Freiburg Center of Data Analysis and Modelling, University of Freiburg, Eckerstr. 1, Freiburg, 79104 Germany

**Keywords:** Homogeneous Markov process, Transition probabilitiy, Attributable mortality, Population-attributable fraction, Nosocomial infection

## Abstract

**Background:**

The extended illness-death model is a useful tool to study the risks and consequences of hospital-acquired infections (HAIs). The statistical quantities of interest are the transition-specific hazard rates and the transition probabilities as well as attributable mortality (AM) and the population-attributable fraction (PAF). In the most general case calculation of these expressions is mathematically complex.

**Methods:**

When assuming time-constant hazards calculation of the quantities of interest is facilitated. In this situation the transition probabilities can be expressed in closed mathematical forms. The estimators for AM and PAF can be easily derived from these forms.

**Results:**

In this paper, we show how to explicitly calculate all the transition probabilities of an extended-illness model with constant hazards. Using a parametric model to estimate the time-constant transition specific hazard rates of a data example, the transition probabilities, AM and PAF can be directly calculated. With a publicly available data example, we show how the approach provides first insights into principle time-dynamics and data structure.

**Conclusion:**

Assuming constant hazards facilitates the understanding of multi-state processes. Even in a non-constant hazards setting, the approach is a helpful first step for a comprehensive investigation of complex data.

**Electronic supplementary material:**

The online version of this article (doi:10.1186/s12874-017-0379-4) contains supplementary material, which is available to authorized users.

## Background

Understanding the correct use of statistical models in hospital epidemiology is challenging. When studying the burden of hospital-acquired infections (HAIs) one has to not only account for the time-dynamic of the acquisition of an HAI but also for competing events (hospital death and discharge).

Different statistical models and approaches are available to study the occurrence, determinants and consequences of HAIs. Pierce et al. [1] nicely contrast and compare these methods, which range from simple logistic regression to complex multi-state models in survival analysis.

While the logistic regression model is the most frequently used model, it has a number of restrictions. As it ignores the timing of the events it is only appropriate for a crude investigation of the data. In contrast, multi-state model approaches adequately account for time-dependencies, but have a complex mathematical background.

In the companion paper [2] to this one, we link the results of a logistic regression to the results of a multi-state model approach based on constant hazard rates. The focus is on time-constant quantities and the basics of multi-state methods are discussed.

**Fig. 1:**
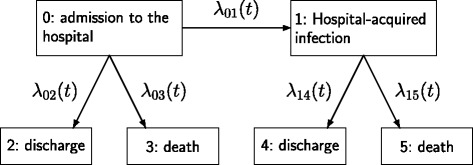
Extended Illness-Death Model with hazard rates *λ*_01_(*t*),*λ*_02_(*t*), *λ*_03_(*t*), *λ*_14_(*t*) and *λ*_15_(*t*)

In this paper, we aim to give a simple explanation of the time-dynamics in a multi-state model approach. We focus specifically on the extended illness-death model (Fig. 1). This multi-state model is a useful tool to study the association of HAIs and mortality in observational cohort studies. It accounts for death in hospital and discharge alive as competing events. In order to account for the time-dependency of the acquisition of infections, the event is modelled as intermediate event. The model allows death and discharge to occur without or after the acquisition of an HAI.

A complete multi-state model analysis includes an investigation of the hazard rates and the transition probabilities [3]. In this paper we discuss these quantities in a time-constant hazards setting. This is the rare setting where the hazard rates are simply accessible, formally obtained via maximum likelihood estimation [4]. Moreover, a derivation of closed mathematical forms for the transition probabilities becomes feasible. We calculate these forms for the extended illness-death model. The transition probabilities are further being used for an analysis of the data in terms of attributable mortality.

With an application to the publicly available dataset *los.data* from the Comprehensive R Archive Network package (R) *etm* we aim to show the performance of the theoretical time-constant quantities in a real application. The data example is a sample from the SIR-3 study, which is an observational cohort study conducted to analyse the burden of HAIs. The data example is best analysed with an extended illness-death model.

We begin in “The extended illness-death model” with a mathematical definition of the extended illness-death model. This section is followed by the derivation of closed mathematical forms for the transition probabilities in a time constant hazards setting. In “Attributable mortality of hospital-acquired infectionsAttributable morta-lity of hospital-acquired infections” we show how the formulas of the transition probabilities, derived in the section “Closed mathematical forms for the transition probabilitiesClosed mathematical forms for the transitionprobabilities”, can be used to estimate the attributable mortality risk of HAIs. The data example is presented in “Results and discussion”. We close in the last section with a discussion.

## Methods

### The extended illness-death model

In hospital-epidemiology the extended illness-death model, shown in Fig. 1, is a useful tool to study HAIs. The model allows both an etiological exploration of the events infection and in-hospital death and an investigation in terms of absolute risks.

In the extended illness-death model patients start in state 0 which is admission to the hospital. At this time none of the patients is infected. The patients remain in this state until they either leave the hospital without an HAI (state 2 if the discharge is alive, state 3 if the patient dies) or acquire an HAI. The concerned patients then move to state 1. In state 1 the patients remain under observation until discharge alive with an HAI (state 4) or death in the hospital with an HAI (state 5). The model is equivalent to the four-state model where death without and with an HAI are one state (state 3 and 5 are modelled as state 3) as well as discharge without and with an HAI (state 2 and 4 are modelled as state 2). In the four-state extended illness-death model a transition from state 1 to state 2 and 3 is possible. The six-state model is a progressive model and has the advantage that it is possible to generally express the transition probabilities even for non-Markov and non-semi-Markov models [5]. Further, we prefer to use the six-state model as it is easier to distinguish and illustrate the transition probabilities without an HAI and those with an HAI.

The extended illness-death model accounts not only for the time-dependency of the acquisition of HAIs, but also differentiates between the competing events discharge alive and death. Wolkewitz et al. [6] explain why it is highly important to account for discharge alive when studying the event hospital-death. As we will see in “Closed mathematical forms for the transition probabilitiesClosed mathe-matical forms for the transition probabilities”, the transition probabilities of the multi-state model depend on all the hazard rates. Ignoring discharge alive would lead to biased estimations.

For a statistical exploration of the data with an extended illness-death model, Andersen et al. [7] propose to describe the model as a stochastic process (*X*(*t*),0≤*t*≤*τ*), where *τ* denotes the end of study time. The stochastic process has right continuous sample paths and a finite state space $S=\bigl \{0,1,2, 3, 4, 5 \bigr \}$.

This mathematical formulation allows an analysis in terms of transition-specific hazards and transition probabilities. The transition probabilities of Markov multi-state models are defined as *P*_*ij*_(*s*,*t*)=*P*(*X*(*t*)=*j*|*X*(*s*)=*i*),0≤*s*≤*t*≤*τ*, *i*,*j*∈*S*. The transition-specific hazard rates, also known as transition intensities, are directly linked to the transition probabilities. They are given by $\lambda _{ij}(t)=\lim \limits _{dt\to 0}\frac {P_{ij}(t,t+dt)}{dt}$, where 0≤*t*≤*τ*, *i*,*j*∈*S*, provided the limits exist. Multi-state models are fully determined by the transition-specific hazard rates [3]. The Markov property implies that the future distribution of the process depends on the present only. For the extended illness-death model this means that the risk of death and discharge given an HAI depends only on the total length of stay in the hospital and not on the length of stay in the hospital until acquisition of an HAI. This assumption can be tested by including time of acquistion of an HAI as covariate in a Cox proportional hazards model for the transitions from state 1 to states 4 and 5 [8].

As a consequence, transition probabilities from state 1 are defined since time origin even though all patients start in state 0, which implies *P*(*X*(0)=1)=0. In the estimation patients entering state 1 are treated as left-truncated. This means they are assumed to be infected since time point zero, but were not observed until time of infection.

In practice the transition probabilities in state 1 become relevant as soon as the first patients acquire an HAI. By definition of HAIs, this is not before day three since admission. Thus, the definition from time point zero to three is rather artificial. In the data application we therefore choose two different landmark time-points for a more practical illustration of the transition probabilities from state 1. This approach corresponds to the conditional survival function discussed for example in Andersen et al. [9].

In the following we assume constant hazard rates. Thus, *λ*_*ij*_(*t*)=*λ*_*ij*_ for *i*∈{0,1} and *j*∈{1,2,3,4,5}. As a consequence, we have $P_{ij}(s,t)=P(X(t)=j|X(s)=i)=P(X(t-s)=j|X(0)=i)=P_{ij}(0,t-s)=P_{ij}(0,\tilde {t})$. The constant hazards model implies that the instantaneous risk of any event in the model is the same throughout the whole hospital stay of a patient. The effect on the transition probabilities is that they only depend on the considered time frame $\tilde {t}=t-s$, but not on the current time point *s*.

Using constant hazards is the most simple parametric model to describe a multi-state process. Maximum likelihood methods are available to obtain estimators for the hazard rates in a real dataset. A formal derivation of the approach for an extended illness-death model can be found in the Appendix. In most situations the constant hazard assumption is too simple. For a profound analysis often more sophisticated parametric and semi- or non-parametric methods are needed (see e.g. [10, 11]). Nevertheless, in this paper and the companion paper [2] we show that assuming constant hazards is an easily accessible way to obtain a fast general understanding of the data.

### Closed mathematical forms for the transition probabilities

Given all the transition-specific hazard rates of a multi-state model, it is theoretically possible to calculate and estimate the transition probabilities. Nevertheless, in non-Markov models this calculation is infeasible. For Markov models the calculation is facilitated as it is possible to use matrix multiplication [3]. Statistical software for an estimation based on a dataset is available [12–14]. However, an explicit mathematical term for a defined set of hazard rates describing a multi-state model is still hard to derive. In contrast, if we assume constant hazards, the transition probabilities of complex multi-state models can have accessible mathematical forms.

In (1) we explicitly formulate the transition probabilities of an extended illness-death model based on arbitrary time-constant hazard rates. An explanation of how we obtain these formulas is given in the Appendix.

With *λ*_0_=*λ*_01_+*λ*_02_+*λ*_03_ and *λ*_1_=*λ*_14_+*λ*_15_, we have

1 $$\begin{array}{@{}rcl@{}} \begin{aligned} P_{00}\left(0, \tilde{t}\right)&=\exp\left(-\lambda_{0}\tilde{t}\right) \\ P_{01}\left(0, \tilde{t}\right)&=\frac{\lambda_{01}}{\left(\lambda_{1}-\lambda_{0}\right)}\left(\exp\left(-\lambda_{0}\tilde{t}\right)-\exp\left(-\lambda_{1}\tilde{t}\right)\right)\\ P_{02}\left(0, \tilde{t}\right)&=\frac{\lambda_{02}}{\lambda_{0}}\left(1-\exp\left(-\lambda_{0}\tilde{t}\right)\right)\\ P_{03}\left(0, \tilde{t}\right)&=\frac{\lambda_{03}}{\lambda_{0}}\left(1-\exp\left(-\lambda_{0}\tilde{t}\right)\right)\\ P_{11}(0, \tilde{t})&=\exp\left(-\lambda_{1}\tilde{t}\right)\\ P_{14}(0, \tilde{t})&=\frac{\lambda_{14}}{\lambda_{1}}\left(1-\exp\left(-\lambda_{1}\tilde{t}\right)\right)\\ P_{15}(0, \tilde{t})&=\frac{\lambda_{15}}{\lambda_{1}}\left(1-\exp\left(-\lambda_{1}\tilde{t}\right)\right)\\ P_{04}(0, \tilde{t})&=\frac{\lambda_{01}\lambda_{14}}{\lambda_{0}\lambda_{1}} \,-\, \frac{\lambda_{01}\lambda_{14}}{\lambda_{0}\left(\lambda_{1}-\lambda_{0}\right)}\exp\!\left(-\lambda_{0}\tilde{t}\right) \,+\, \frac{\lambda_{01}\lambda_{14}}{\lambda_{1}\left(\lambda_{1}-\lambda_{0}\right)}\!\exp\!\left(\!-\lambda_{1}\tilde{t}\right)\!\!\!\!\!\!\!\!\!\!\!\!\!\!\!\!\!\!\!\!\!\!\!\!\!\!\!\!\\ P_{05}(0, \tilde{t})&=\!\frac{\lambda_{01}\lambda_{15}}{\lambda_{0}\lambda_{1}} \,-\, \frac{\lambda_{01}\lambda_{15}}{\lambda_{0}\left(\lambda_{1}-\lambda_{0}\right)}\exp\!\left(-\lambda_{0}\tilde{t}\right) \,+\,\! \frac{\lambda_{01}\lambda_{15}}{\lambda_{1}\left(\lambda_{1}-\lambda_{0}\right)}\!\exp\!\left(\!-\lambda_{1}\tilde{t}\right)\!,\!\!\!\!\!\!\!\!\!\!\!\!\!\!\!\!\!\!\!\!\!\!\! \end{aligned} \end{array} $$

where $\tilde {t}=t-s$ for any 0≤*s*<*t*. If *λ*_0_=*λ*_1_ then transition probabilities become

$$\begin{array}{*{20}l} P_{01}(0,\tilde{t})&=\lambda_{01}t\exp(-\lambda_{1}\tilde{t})\\ P_{04}(0,\tilde{t})&=\frac{\lambda_{14}\lambda_{01}}{\lambda_{0}\lambda_{1}}(1-\exp(-\lambda_{0}\tilde{t})-\lambda_{0}t\exp(-\lambda_{1}\tilde{t}))\\ P_{05}(0,\tilde{t})&=\frac{\lambda_{15}\lambda_{01}}{\lambda_{0}\lambda_{1}}(1-\exp(-\lambda_{0}\tilde{t})-\lambda_{0}t\exp(-\lambda_{1}\tilde{t})). \end{array} $$

The other transition probabilities remain the same as in the general case *λ*_0_≠*λ*_1_.

These formulas reveal how the various transition probabilities depend on the different transition-specific hazard rates. Furthermore, it is possible to directly obtain the transition probabilities when the hazard rates are known. Thus, complicated estimation based on the data is not necessary in a time-constant hazards setting.

These formulas show, that the transition probabilities from state 0 to state 2 and 3 are independent of the hazard rates after an infection. Nevertheless, they depend on all the three hazards that are modelled out of state 0. The state occupation probability of state 0 also depends on these hazard rates. The transition probabilities from state 1 only depend on the discharge and death hazard after an HAI. Only the transition probabilities from state 0 to states 4 and 5 depend on all the five hazards.

The formulas show that all the transition probabilities depend on time. For *t* towards infinity the limits exist. They are shown in (2).

2 $$\begin{array}{*{20}l} P_{00}(t)& \rightarrow 0\\ P_{01}(t)& \rightarrow 0\\ P_{02}(t)& \rightarrow \lambda_{02}/\lambda_{0}\\ P_{03}(t)& \rightarrow \lambda_{03}/\lambda_{0}\\ P_{11}(t)& \rightarrow 0 \\ P_{14}(t)& \rightarrow \lambda_{14}/\lambda_{1}\\ P_{15}(t)& \rightarrow \lambda_{15}/\lambda_{1}\\ P_{04}(t)& \rightarrow (\lambda_{01}\lambda_{14})/(\lambda_{0}\lambda_{1}) \\ P_{05}(t)& \rightarrow (\lambda_{01}\lambda_{15})/(\lambda_{0}\lambda_{1}), \\ \\ \text{for}~t&\rightarrow \infty. \end{array} $$

The speed of convergence depends on exp(−*λ*_0_ *t*) and exp(−*λ*_1_ *t*). The limits are the proportions of patients that died and were discharged without and with infection at the end of follow-up. Thus, they can be seen as estimators for the probabilities to be in either of the states at the end of the hospital stay. The difference in the limit of moving from state 1 to states 4 and 5 compared to from state 0 lies in conditioning on conditioning that patients are already infected.

### Attributable mortality of hospital-acquired infections

Schumacher et al. [15] show how the transition probabilities of an extended illness-death model can be used to obtain estimators for the attributable mortality (AM) of an HAI and the population attributable fraction (PAF). These two statistical quantities describe the risk increase of hospital death due to the HAI. The attributable mortality is defined as

$$AM(t)=P(D(t)=1|E(t)=1)-P(D(t)=1|E(t)=0), $$

where *D*(*t*) indicates if the patient died by time *t* and *E*(*t*) if an HAI was acquired by time *t*.

If AM(t) is greater than zero, patients that acquire an HAI up to time *t* have a higher death risk than patients that don’t acquire an infection until then. The higher AM(t) the higher is the risk of death for infected patients compared to patients without infection. If AM(t) is smaller than zero, then uninfected patients have a higher risk of death.

The PAF is defined as

$$PAF(t)=\frac{P(D(t)=1)-P(D(t)=1|E(t)=0)}{P(D(t)=1)}. $$

It indicates to what amount the overall hospital mortality would decrease, if there were no HAIs. The resulting fraction is similarly interpreted as *A* *M*(*t*). If *P* *A* *F*(*t*) is greater than zero, the occurrence of HAIs up to time *t* increases the risk of death and therefore the overall death risk. The closer *P* *A* *F*(*t*) is to one, the higher is the attribution of death due to an HAI to the overall death risk at time *t*.

Given the formulas proposed by Schumacher et al. for infections that were only acquired *after* hospital admission (*P*(*X*(0)=0)=1 and *P*(*X*(0)=1)=0), we can calculate by using (1) that

3 $$\begin{array}{*{20}l} P(D(t)&=1|E(t)=1)=\frac{P_{05}(t)}{P_{01}(t)+P_{04}(t)+P_{05}(t)}\\ &=\frac{\lambda_{15}}{\lambda_{1}(\lambda_{1}-\lambda_{0})} \left\{\frac{\lambda_{1}\!\,-\,\lambda_{0}\,-\,\lambda_{1}\exp(\,-\,\lambda_{0} t)\,+\,\lambda_{0}\exp(\,-\,\lambda_{1} t)}{1-\exp(-\lambda_{0} t)}\right\} \end{array} $$

and

4 $$\begin{array}{*{20}l} P(D(t)=1|E(t)=0)&=\frac{P_{03}(t)}{P_{00}(t)+P_{02}(t)+P_{03}(t)}\\ &=\frac{\lambda_{03}(1-\exp(-\lambda_{0} t))} {\lambda_{01}\exp(-\lambda_{0} t)+\lambda_{02}+\lambda_{03}}. \end{array} $$

The overall hospital mortality is the sum of the death risk without and with an HAI among all initially uninfected patients. Thus,

5 $$ P(D(t)=1)=P_{03}(t)+P_{05}(t).   $$

Note that in this setting *t* should be interpreted as time since hospital admission. Above, as we are in a time-constant hazards setting, *t* (denoted as $\tilde {t}$) could be seen as any time-window starting from an arbitrary time-point *s* since admission. This matter is explained in “The extended illness-death model”.

The formulas show that the risk of death given no HAI depends only on the hazard rates without infection. In contrast, the risk of death given an HAI depends on all five hazards. Moreover, there is a clear difference between *P*_03_(*t*) and *P*(*D*(*t*)=1|*E*(*t*)=0) and between *P*_05_(*t*) and *P*(*D*(*t*)=1|*E*(*t*)=1).

*P*_05_(*t*) is the probability to die by time *t* with an infection for an initially uninfected patient. *P*(*D*(*t*)=1|*E*(*t*)=1) is *P*_05_(*t*) devised by the probability of having acquired an infection by time *t*. This probability is the sum of *P*_01_(*t*), *P*_04_(*t*) and *P*_05_(*t*) (in the data example the sum of the lower three graphs in Fig. 3), which is the cumulative incidence function of the acquisition of an infection. In other words, *P*(*D*(*t*)=1|*E*(*t*)=1) is the proportion of patients that die with infection by time *t* among patients with an infection at time *t*.

**Fig. 2:**
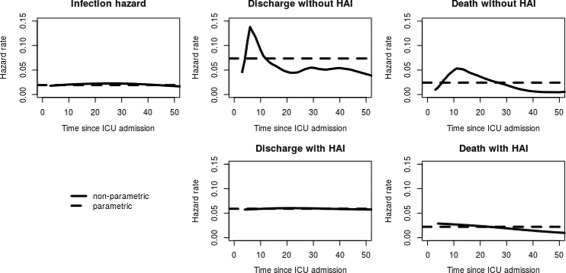
Hazard rates of los.data estimated with non-parametric (*straight line*) and parametric (*dotted line*) methods

Similarly, *P*_03_(*t*) is the probability to die by time *t* without infection for an initially uninfected patient. In other words, it is the proportion of patients that die without an infection by time *t*. *P*(*D*(*t*)=1|*E*(*t*)=0) conditions on being infection-free until time *t*. This condition can be expressed as the converse probability of the acquisition of an infection. Thus, *P*(*D*(*t*)=1|*E*(*t*)=0) is the proportion of all uninfected patients at time *t* that die by time *t*.

Taking the limits of Eqs. (3), (4) and (5), we get

6 $$\begin{array}{*{20}l} P(D(t)=1|E(t)=0) &\rightarrow \frac{\lambda_{03}}{\lambda_{02}+\lambda_{03}}\\ P(D(t)=1|E(t)=1) &\rightarrow \frac{\lambda_{15}}{\lambda_{1}}\\ P(D(t)=1) &\rightarrow \frac{\lambda_{03}\lambda_{1}+\lambda_{01}\lambda_{15}}{\lambda_{0}\lambda_{1}}\\  \\ \text{for}~t &\rightarrow \infty.  \end{array} $$

Interestingly, the limit of the probability that a hospital stay with no HAI ends in death is independent of the infection hazard. Moreover, the limits of *P*_15_(*t*) and *P*(*D*(*t*)=1|*E*(*t*)=1) are the same. *P*_15_(*t*) is the proportion of *all* infected patients that die by time *t*. In contrast to *P*_15_(*t*), *P*(*D*(*t*)=1|*E*(*t*)=1) takes into account that all patients were initially uninfected. For *t* towards infinity eventually all patients that ever had an infection are observed to have acquired the infection.

The limits of AM(t) and PAF(t) are

7 $$\begin{array}{*{20}l} AM(t) &\rightarrow \frac{\lambda_{02}\lambda_{15}-\lambda_{03}\lambda_{14}}{\lambda_{1}\left(\lambda_{02}+\lambda_{03}\right)}\\ PAF(t) &\rightarrow \frac{\lambda_{01}\left(\lambda_{02}\lambda_{15}-\lambda_{03}\lambda_{14}\right)}{\left(\lambda_{02}+\lambda_{03}\right) \left(\lambda_{03}\lambda_{1}+\lambda_{01}\lambda_{15}\right)}\\  \\ \text{for}~t &\rightarrow \infty.  \end{array} $$

The limit of AM(t) is independent of the infection hazard *λ*_01_. The probability of being infected and still in the hospital for *t*→*∞* is 0. The fraction $\frac {\lambda _{02}}{\lambda _{02}+\lambda _{03}}$ is the probability that the patient was discharged at the end of the hospital stay, given he didn’t acquire an HAI. This fraction is multiplied by the end-of-stay probability that the patient died, given he had an HAI. This part of the difference represents the negative impact of an HAI. From this quantity the probability that the opposite happens (death given no HAI, discharge given an HAI) is subtracted. Moreover, the limits are exactly the same number we would get, when disregarding the time of acquisition of an HAI.

PAF(t) converges to a value that also depends on the infection hazard. As the PAF is a measure for the overall mortality risk, the quantity depends on the amount of patients that acquire an HAI. If the infection is rare, then the overall mortality risk is not as much increased as for a large infection rate, given the same mortality and discharge rates.

In the companion paper, the limits (2) and (7) are interpreted and linked to the results of a logistic regression analysis.

## Results and discussion

In this section, we demonstrate how to apply the formulas from the sections “Closed mathematical forms for the transition probabilitiesClosed mathematical forms forthe transition probabilities” and “Attributable mortality of hospital-acquired infectionsAttributable mortalityof hospital-acquired infections” to a real data example. We use the publicly available *los.data* from the R-package *etm*. The data is a sample from the SIR-3 cohort study. This is an observational cohort study from the Charité university hospital in Berlin, Germany. The data was prospectively accessed to examine the effect of HAIs in intensive care (ICU) [16]. The study followed 756 patients over the time period from February 2000 until July 2001. Of these patients 475 patients were discharged alive without an HAI and 157 died without an HAI. Of the 124 patients that acquired an HAI, 90 were discharged and 34 died. In this sample of the SIR-3 cohort study none of the patients were censored.

We first use non-parametric Markov methods to estimate the transition specific hazard rates and the transition probabilities. The non-parametric hazard rates are estimated with the R-package *bshazard* [17]. This package uses B-splines to obtain smooth estimators of all transition-specific hazards based on the data sample. In order to estimate the transition probabilities non-parametrically, we use the R-package *etm* [12]. This package uses matrix multiplication to obtain the Aalen-Johansen estimators for all possible transition probabilities in a multi-state Markov model. A detailed description is given in the tutorial by Allignol et al. [12].

In order to apply the closed mathematical forms of the transition probabilities, we further estimate the constant hazard rates based on the data. The maximum likelihood estimator of each transition specific hazard is simply the occurrence/exposure rate [4], given by

8 $$ \hat{\lambda}_{ij}=\frac{\text{Number of {i} \(\rightarrow\) {j} transitions}}{\text{Summed patient-days in state {i}}}.   $$

These are the time-constant counterparts of the non-parametric transition-specific hazard rates of the data. A formal derivation of the parametric estimators is provided in the Appendix.

Figure 2 shows the non-parametric transition-specific hazard rates with their parametric counterparts that are based on (8). The figure shows that the time-constant parametric hazards roughly approximate the bshazard-based ones. While the infection hazard as well as the discharge and death hazard after an HAI seem to be more or less constant, this is not the case for the discharge and death hazards without an HAI.

**Fig. 3:**
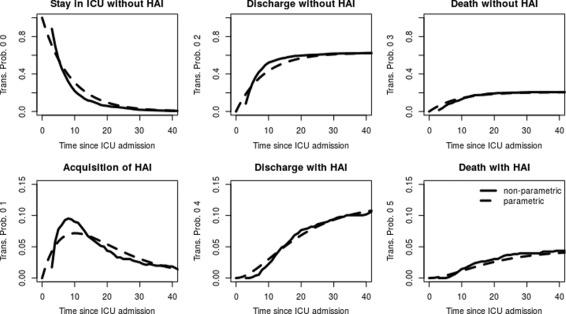
Transition probabilities from state 0 of los.data estimated with non-parametric (*straight line*) and parametric (*dotted line*) methods

**Fig. 4:**
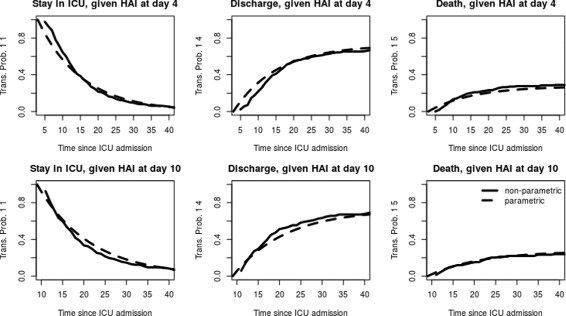
Transition probabilities from state 1 of los.data estimated with non-parametric (*straight line*) and parametric (*dotted line*) methods at the landmark time points 4 and 10 days after admission

In a next step the time-constant hazard rates are plugged into Eq. (1) to obtain the transition probabilities. Figures 3 and 4 show the resulting curves next to the transition probabilities that are based on an estimation with *etm*. In Fig. 3 the transition probabilities from state 0 are illustrated.Since all patients start in state 0 at time zero, we estimate and show the graphs from time origin. The plots show that the probability to stay in the ICU quickly decreases while the probability of discharge and death without an HAI increase. The probability of discharge without an HAI is clearly larger than the death risk. The probability to acquire an HAI and still being an inpatient is a bit lower and reaches a peak around day 10. The risk of first acquiring an infection and then dying or being discharged is also quite low due to the relatively low infection risk.

The plots further show that the parametric estimators behave in the same way as the non-parametric ones. Despite the circumstance that not all the transition-specific hazard rates seem to be constant, the transition probabilities of the two approaches appear quite similar. Though there are observable discrepancies. These are mainly due to the discharge hazard which is not constant. Patients are more likely to be discharged after about seven to ten days since admission than on other days. Moreover, this hazard has the strongest impact as most of the patients are discharged without an HAI.

Figure 4 shows the transition probabilities from state 1 for two different landmark time points (days four and ten after ICU admission). Since we assume a Markov model, the transition probabilities in state 1 are defined since time origin. Nevertheless, as an HAI can’t occur before three days after admission per definition, no events happen before day four. Therefore, in practice the transition probabilities become more relevant at later time-points. Hence, we have chosen to estimate the transition probabilities at the landmarks four and ten.

The parametric approach is independent of the starting time point *s*. Accordingly, the transition probabilities estimated with the formulas are the same for both landmarks. In the non-parametric approach we observe slight differences between the landmarks. The estimators at landmark four are the same as those at time-origin zero. At landmark ten, the probability of staying in the ICU with an HAI seems to be slightly decreased compared to landmark four. In contrast, the discharge chances seem to be increased while the death risk is also slightly decreased. These observed discrepancies arise mainly from the death hazard with HAI which is slightly decreasing. Nevertheless, these differences between the two landmarks are minor and the parametric estimators nicely approximate their non-parametric counterparts at both landmarks.

**Fig. 5:**
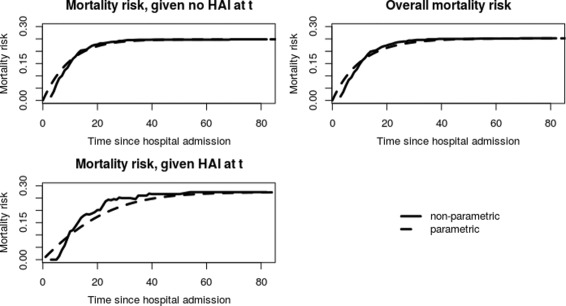
Mortality risks in los.data for uninfected and infected patients, as well as the overall risk estimated with non-parametric (*straight lines*) and parametric (*dotted lines*) methods

**Fig. 6:**
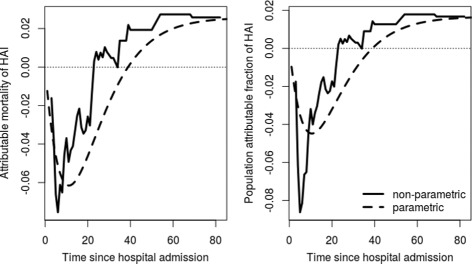
Attributable mortality and population attributable fraction of HAIs for los.data estimated with non-parametric (*straight lines*) and parametric (*dotted lines*) methods

Furthermore, we used the time-constant hazard rates as plug-in values in (3) to (5) to obtain parametric estimators for AM and PAF. In Fig. 5 we see the overall hospital mortality, the mortality risk given no HAI and the mortality risk given an HAI. Figure 6 shows the resulting AM and PAF. The plots show both, the parametric and the non-parametric, estimators.

The figures demonstrate that both, the mortality risks and the quantities AM and PAF, are roughly approximated by their parametric analogous. The limits are the same.

The overall mortality and the mortality given that no infection occurs, shown in Fig. 5, increase strongly up to 40 days after admission to the ICU and then both converge to ∼0.25. Thus, past 40 days the overall hospital mortality and hospital mortality given that no infection occurs until that time is approximately 25%. The two curves show no big difference. This may either imply that the mortality risk among already infected patients is not very different to that of still uninfected patients or that the infection risk is very low. Then, infected patients have not a great influence on the overall mortality risk. In our case, we have a comparable low infection risk, but also the risk of death for already infected patients is not highly increased after an infection as can also be seen in Fig. 5.

The risk of death given that an infection occurred constantly increases until 60 days after admission and then converges to ∼0.28. The risk of death given an HAI is lower than the overall mortality and the mortality without an HAI until about 40 days after admission. The reason for this is that patients have to make a transition from state 0 to state 1 first in order to be at risk of dying with an HAI. Thus, events in the group of infected patients are delayed. We would also like to point out, that infected patients are observed to die more frequently in the ICU due to a decreased discharge hazard. While the death hazards before and after an infection are quite similar, the discharge hazard with an HAI is clearly lower than without an HAI. Thus, the discharge hazards affect the length of stay in the ICU. Patients with an HAI stay longer in the ICU and are therefore more often observed to die in the ICU.

As a consequence, AM and PAF are smaller than zero in the first 40 days. A plateau is reached about 60 days after admission. At the end of the hospital stay the AM is ∼0.026 and the PAF is ∼0.017. We obtain the same numbers with Eq. (7) and the time constant hazard rates estimated with (8).

There are apparent differences between the parametric and the non-parametric approach, especially observable in Fig. 6. These differences are consequences of the differences in the transition-probabilities which were in this example mainly due to the non-constant discharge hazard without HAI.

The complete R code of this analysis is provided in the Additional file 1. The functions to calculate the parametric transition probabilities can be used for any data example that corresponds to an extended illness-death model.

## Conclusions

In this paper closed mathematical forms for the transition probabilities of an extended illness-death model are derived. Despite the complexity of the mathematical processes behind the multi-state model, the terms are accessible for constant hazard rates. Multi-state models depend on all the hazard rates and additionally on time. In this paper we reveal the mechanisms underlying an extended illness-death model with constant hazards. Moreover, the derived formulas can be used to perform multi-state analysis in a highly facilitated manner by using the time-constant hazard rates as plug-in values. These are easily obtained by dividing the number of observed events by the total number of patient days (uninfected or respectively infected). Thus, no sophisticated methods are needed to obtain a first insight in the determinants and consequences of HAIs using multi-state methodology.

The application to a specific data example shows that the results obtained with the time-constant hazards analysis show the same time-dynamics as those of the non-parametric Markov methods for multi-state models. The data is a sample of the SIR-3 cohort, which is a German observational study from 2001 to access information about HAIs and the association to hospital mortality. We use the R-package *etm* to estimate the transition probabilities. The R-function assumes that the sample fulfils the Markov assumption. Testing this assumption in the way described in “The extended illness-death model”, we obtain non-significant hazard ratios for the time of an HAI. Therefore, we presume that the Markov assumption is adequate. Note that the test we use only allows a rejection of the Markov assumption, but does not prove it’s plausibility. The non-parametric hazard rates are estimated with the R-package *bshazard*.

A comparison of the non-parametric estimators of the hazard rates to the parametric ones shows that the infection hazard as well as the discharge and death hazard with an HAI are more or less in concordance with the time-constant ones. The discharge hazard and the death hazard without an HAI are clearly not constant. Despite these violations of the constant hazards assumption, all the transition probabilities obtained with the mathematical terms show high concordance to the non-parametric ones. We have made the same experience with larger datasets on HAIs from France and Spain. Nevertheless, we recommend to use this parametric approach only as a first step in the data analysis. If the constant hazards assumption is clearly violated, the resulting estimators offer only insights into basic data structure and time-dynamics. For a profound understanding more sophisticated methods are needed.

We further use the transition probabilities to calculate the AM and the PAF of HAIs. We present closed mathematical forms for the risk of death depending on the state of infection in the course of time. Together with the overall mortality risk, these are the components of the AM and the PAF. In the data example these mathematical terms are compared to the non-parametric ones. The mathematical forms roughly approximate the results from the non-parametric estimation. The limits of the estimators are exactly the same and correspond to an estimation where time of an HAI and infection is completely neglected [2].

The R-code for the data analysis can be found in the Additional file 1. We further implemented a function to calculate the parametric transition probabilities for any extended illness-death model. The five transition-specific hazard rates are used as plug-in values and can be obtained from any dataset of interest. Hence, the R code can be used to perform a parametric multi-state analysis or to perform a re-analysis of reports if the five hazard rates are available from the publication.

As a consequence, we propose that all five hazards (or the total number of patient days (infected and uninfected) as well as the observed number of each event) should always be published. That way the reader has the chance to retrace the analysis in a simplified setting (see also Wolkewitz et al. [2]).

In the companion paper [2] we discuss the multi-state analysis with constant hazards in a more basic manner. The extended illness-death model can be used for example to estimate the extra length of hospital stay due to HAIs [18].

The proposed approach has clear limitations as the constant hazards assumption is often not met in practice. Then, the obtained estimators may be misspecified. In this case, we recommend to use this parametric approach only as a first step in the data analysis. Semi- and non-parametric approaches are often more suitable for a correctly specified data analysis. Especially when the Markov assumption is met, a great variety of easily applicable statistical software is available [10]. Moreover, we have not derived closed mathematical forms for the variances of the parametric transition probabilities. For a more comprehensive investigation of the data, an estimation of the deviation from the obtained estimators is essential.

Nevertheless, assuming the highly facilitated setting of time-constant event rates is sufficient for a first understanding of the data. We recommend this approach to gain a basic understanding of the data and the statistical quantities of interest. We hope this simplified approach leads to a better understanding of multi-state processes and is easier to communicate to non-statisticians.

The use of these methods has a wide range of applications. The extended illness-death model is a general concept that may be used to study any data setting with adverse outcomes and a binary time-dependent covariate.

## Appendix: Calculation of the transition probabilities for a Multi-state model with constant hazards

### Short introduction of the general theory

In order to calculate the transition probabilities of an extended illness-death model with constant hazards we follow the book of Hougaard [19]. For a deeper understanding also for general Markov multi-state models we refer to Kijima [20] and Aalen et al. [3].

The big advantage of Markov models is the possibility to obtain the transition probabilities at a time-point *t* via Matrix multiplication. Is the model furthermore time-homogeneous, only the initial distribution and the one-step transition matrix is needed for a calculation. In a time-discrete model, this means *P*(*n*)=*P* ^*n*^, where *P* is the one-step transition matrix and *n* is the *n*-th step. The one-step transition matrix has entries *p*_*ij*_=*P*(*X*(*n*+1)=*j*|*X*(*n*)=*i*)=*P*(*X*(1)=*j*|*X*(0)=*i*) [3].

For a time-continuous Markov model the argumentation is more complex. In order to obtain the transition probabilities at a time point *t*>0, we need to define the so-called infinitesimal generator ***λ***. The off-diagonal elements of this matrix are the transition intensities (the transition-specific hazard rates) of the multi-state model. The diagonal elements are the negative total hazards (the sum of the hazards from the initial and transient states). Thus, each row of the matrix add up zero. The relationship of *P* and ***λ*** leads to

9 $$ \frac{dP(t)}{dt}=P(t)\boldsymbol{\lambda},   $$

where *P*(*t*)=(*P*_*ij*_(0,*t*))_*i*,*j*∈*S*_ is the transition probability matrix at time *t*. This equation follows from the Chapman-Kolmogorov equations. The initial condition is *P*(0)=***I***, where ***I*** is the identity matrix [3]. Remark that $\boldsymbol {\lambda }=\frac {dP(0)}{dt}$. A solution of this equation is given by

10 $$ P(t)=\exp(\boldsymbol{\lambda}t)=\sum_{n=0}^{\infty}\frac{(\boldsymbol{\lambda}t)^{n}}{n!}.   $$

If ***λ*** has distinct Eigenvalues, than the matrix is diagonalizable and we have

$$\begin{array}{*{20}l} P(t)&=\exp(\boldsymbol{\lambda}t)=\sum_{n=0}^{\infty}\frac{(\boldsymbol{\lambda}t)^{n}}{n!}\\ &=M\sum_{n=0}^{\infty}\frac{(\boldsymbol{\lambda_{D}}t)^{n}}{n!}M^{-1}\\ &=M\exp(\boldsymbol{\lambda_{D}}t)M^{-1}, \end{array} $$

where ***λ***_***D***_ is a diagonalmatrix and *M* is a regular transformation matrix. In this case the transition probabilities can be written as the sum of exponential equations. The Eigenvalues are the exponents. In case where some Eigenvalues have multiplicity higher than one, there exists a Jordan-Normalform for the Matrix ***λ***. The transition probabilities can then be expressed as combined polynomials and exponential functions [19].

### The transition probabilities of an extended illness-death model

In this section we explicitly show how to calculate the transition probabilities of an extended illness-death model (shown in Fig. 1). The time-constant hazard rates of the model are denoted *λ*_01_, *λ*_02_, *λ*_03_, *λ*_14_, and *λ*_15_. The total hazard is defined as the sum over all hazard rates modelled from a specific state. In the extended illness-death model the total hazards are *λ*_0_=*λ*_01_+*λ*_02_+*λ*_03_ from state 0 and *λ*_1_=*λ*_14_+*λ*_15_ from state 1.

According to Hougaard [19] the transition probabilities of this model can be written as

11 $$ P_{ij}(0,t)=\alpha_{ij1}\exp(-\lambda_{0} t)+\alpha_{ij2}\exp(-\lambda_{1} t)+\alpha_{ij3}.   $$

The values *λ*_0_, *λ*_1_ and 0 are the eigenvalues of the 6×6 matrix ***λ***. This is a matrix with the diagonal elements ***λ***_00_=−*λ*_0_, ***λ***_11_=−*λ*_1_ and ***λ***_*ii*_=0 for *i*=2,3,4,5. The off-diagonal elements are the transition-specific hazard rates ***λ***_*ij*_=*λ*_*ij*_ if the transition exits, 0 otherwise.

To correctly solve the Eq. (11) under the conditions imposed by (9), Hougaard introduces boundary and balance equations. The boundary equations are given by

12 $$ \sum_{r} \alpha_{iir}=1, \quad \sum_{r} \alpha_{ijr}=0, i \neq j,   $$

for *r*=1,2,3. The balance equations are

13 $$ -\alpha_{ijr}\beta_{r}=\sum_{k=0}^{5}\alpha_{ikr}\lambda_{ik},   $$

where *β*_*r*_ (*r*=1,2,3) are the Eigenvalues of ***λ***. Solving the Eqs. (12) and (13) for each combination of *i*=0,1 and *j*=1,2,3,4,5, we obtain the transition probabilities of the extended illness-death model.

In the following we provide a simplified way to obtain the transition probabilities. It is possible to apply the same trick as used by Hougaard in [19] to calculate the transition probabilities of an illness-death model. That is, we define the reduced matrix $\boldsymbol {\lambda }_{red}=\begin {array}{cc} -\lambda _{0} & \lambda _{01} \\ 0 & -\lambda _{1} \end {array}$of a hidden Markov model. The Eigenvalues of this matrix are *λ*_0_ and *λ*_1_. This reduction facilitates the calculation of *P*_00_(0,*t*),*P*_01_(0,*t*) and *P*_11_(0,*t*). After solving the boundary and balance equations we obtain

$$\begin{array}{*{20}l} P_{00}(0,t)&=\exp(-\lambda_{0}t)\\ P_{11}(0,t)&=\exp(-\lambda_{1}t)\\ P_{01}(0,t)&=\frac{\lambda_{01}}{(\lambda_{1}-\lambda_{0})}(\exp(-\lambda_{0}t)-\exp(-\lambda_{1}t)) \end{array} $$

Now we extend ***λ***_*red*_ by including the hazard rates *λ*_02_ and *λ*_03_. We obtain a 6×6-matrix with the Eigenvalues *λ*_0_, *λ*_1_ and 0. By the boundary and balance equations it is easily calculated that

$$\begin{array}{*{20}l} P_{02}(0,t)&=\frac{\lambda_{02}}{\lambda_{0}}(1-\exp(-\lambda_{0}t)),\\ P_{03}(0,t)&=\frac{\lambda_{03}}{\lambda_{0}}(1-\exp(-\lambda_{0}t)). \end{array} $$

Further, we note that a Markov model is a “nested series of competing risks” [10]. Therefore, we have a simple competing risks situation (with left-truncated entry times) in state 1. At each time point *t* we have *P*_14_(0,*t*)+*P*_15_(0,*t*)=1−*P*_11_(0,*t*). The transition probability *P*_14_(0,*t*) is given by the probability 1−*P*_11_(0,*t*) times the probability that the individual ends up in state 4 and not in state 5. This corresponds to a Bernoulli-experiment with probability of success $\frac {\lambda _{14}}{\lambda _{1}}$ that the state is 4. Analogously, for *P*_15_(0,*t*) we multiply by $\frac {\lambda _{15}}{\lambda _{1}}$. Thus,

$$\begin{array}{*{20}l} P_{14}(0,t)&=\frac{\lambda_{14}}{\lambda_{1}}(1-P_{11}(0,t))=\frac{\lambda_{14}}{\lambda_{1}}(1-\exp(-\lambda_{1}t))\\ P_{15}(0,t)&=\frac{\lambda_{15}}{\lambda_{1}}(1-P_{11}(0,t))=\frac{\lambda_{15}}{\lambda_{1}}(1-\exp(-\lambda_{1}t)) \end{array} $$

Finally, we calculate *P*_04_(0,*t*) and *P*_05_(0,*t*) by using *P*_04_(0,*t*)+*P*_05_(0,*t*)=1−(*P*_00_(0,*t*)+*P*_01_(0,*t*)+*P*_02_(0,*t*)+*P*_03_(0,*t*)). Analogous to the argument above, we have

$$\begin{aligned} P_{04}(0,t)&=\frac{\lambda_{14}}{\lambda_{1}}(1\!\!-P_{00}(0,t)-P_{01}(0,t)-P_{02}(0,t)-P_{03}(0,t))\\ &=\!\!\frac{\lambda_{01}\lambda_{14}}{\lambda_{0}\lambda_{1}} \!\,-\, \frac{\lambda_{01}\lambda_{14}}{\lambda_{0}(\lambda_{1}-\lambda_{0})}\!\exp(\!-\lambda_{0}t)\! +\! \frac{\lambda_{01}\lambda_{14}}{\lambda_{1}(\lambda_{1}\!\!-\lambda_{0})}\!\exp(\!-\lambda_{1}t)\\ P_{05}(0,t)&=\!\!\frac{\lambda_{15}}{\lambda_{1}}(1-P_{00}(0,t)-P_{01}(0,t)-P_{02}(0,t)-P_{03}(0,t))\\ &=\!\!\frac{\lambda_{01}\lambda_{15}}{\lambda_{0}\lambda_{1}} \!\,-\, \frac{\lambda_{01}\lambda_{15}}{\lambda_{0}(\lambda_{1}\!-\lambda_{0})}\!\exp(\!-\lambda_{0}t)\! +\! \frac{\lambda_{01}\lambda_{15}}{\lambda_{1}(\lambda_{1}\,-\,\lambda_{0})}\!\exp(\!-\lambda_{1}t). \end{aligned} $$

## Appendix: Maximum likelihood estimation of the constant hazard rates

Time-constant hazard rates imply that the event times follow an exponential distribution. Explicitly for the extended illness-death model (figure ??), this means that in state 0 an event-time *T* is assumed to be generated by exp(*λ*_0_) and in state 1 by exp(*λ*_1_), where *λ*_0_=*λ*_01_+*λ*_02_+*λ*_03_ and *λ*_1_=*λ*_14_+*λ*_15_. The type of event is generated by $\frac {\lambda _{ij}}{\lambda _{i}}$ for *i*=0,1, *j*=1,2,3,4,5 and *i*≠*j*. Note that *λ*_*ij*_=0 for *i*=0 and *j*=4,5 and also for *i*=1 and *j*=2,3.

The parameters we want to estimate with our dataset are the five transition-specific hazard rates. These can be obtained by standard maximum likelihood methods. A general derivation of maximum likelihood methods in survival and event history analysis is given by Andersen et al. [21]. A more explicit but less detailed description of maximum likelihood methods for parametric hazards estimation of multi-state models is formulated by Andersen and Perme [4]. In the special case of the extended illness-death model, the maximum likelihood function is

$$\begin{aligned}L(\lambda_{01},\lambda_{02},\lambda_{03},\lambda_{14},\lambda_{15})&=\prod_{h=1}^{n}\prod_{i,j\in S\atop i\ne j}\left(\prod_{t\leq\tau} \lambda_{ijh}(t)^{\Delta N_{ijh}(t)}\right)\\ &\times \exp\left(-\int_{0}^{C_{h}}\lambda_{ijh}(u)du\right), \end{aligned} $$

where *n* is the total number of individuals in the dataset, *S*={0,1,2,3,4,5} is the state space, *τ* is the endpoint of the study time and *C*_*h*_≤*τ* is the of the event times independent right-censoring time of individual *h*. Further, *N*_*ijh*_(*t*) is the number of transitions of individual *h* from state *i* to state *j* in [0,*t*] and *Δ* *N*_*ijh*_(*t*) is the number of transitions at the time point *t*. With *λ*_*ijh*_(*t*)=*λ*_*ij*_ *Y*_*ih*_(*t*) we denote the individual hazard rate from state *i* to state *j* of patient *h*. The function $\phantom {\dot {i}\!}Y_{ih}(t)=\mathbf {1}_{\{T_{ih}>t\}}$ indicates if the exit time *T*_*ih*_ from state *i* of individual *h* is greater than *t*. In order to obtain the maximum likelihood estimator we use the standard procedure. First the derivative of the logarithm of *L* is calculated. The log likelihood is

$$\begin{aligned} \log\left(L\left(\lambda_{01},\lambda_{02},\lambda_{03},\lambda_{14},\lambda_{15}\right)\right) &=\sum_{h=1}^{n}\sum_{i,j\in S\atop i\ne j}\left(\sum_{t\leq\tau} \log(\lambda_{ijh}(t))\Delta N_{ijh}(t)\right)\\ & \quad -\lambda_{ij}\left(T_{ih}\wedge C_{h}\right). \end{aligned} $$

Then, the score statistic of each *λ*_*ij*_≠0,*i*≠*j* is derived. For one parameter *λ*_*ij*_ it is given by

$$\begin{array}{*{20}l} \frac{\partial log(L)}{\partial \lambda_{ij}}&=\sum_{h=1}^{n}\sum_{t\leq\tau}\frac{\Delta N_{ijh}(t)}{\lambda_{ijh}(t)}-\sum_{h=1}^{n}(T_{ih}\wedge C_{h})\\ &=\lambda_{ij}\sum_{h=1}^{n}\sum_{t\leq\tau}\frac{\Delta N_{ijh}(t)}{Y_{ih}(t)}-\sum_{h=1}^{n}(T_{ih}\wedge C_{h}). \end{array} $$

Setting this to zero and solving for the parameter *λ*_*ij*_ we get as maximum likelihood estimator

$$\hat{\lambda}_{ij}=\frac{\sum_{h=1}^{n}\sum_{t\leq\tau}\frac{\Delta N_{ijh}(t)}{Y_{ih}(t)}}{\sum_{h=1}^{n}\left(T_{hi}\wedge C_{h}\right)}=\frac{N_{ij}}{D_{i}}, $$

where *N*_*ij*_ is the total number of observed transitions from state *i* to state *j* and *D*_*i*_ is the total number of patient-days in state *i*. Thus, the maximum likelihood estimator of the constant hazards in a multi-state model are the basic “occurrence/exposure rates” [4].

## Additional files

Additional file 1 R function to calculate transition probabilities. This is the R-function used in the data analysis (Additional file 2) to calculate the transition probabilities of an extended illness-death model with constant hazards as plug-in values. (R 3 kb)

Additional file 2 R code of analysis of data example. This is the complete R script used in section 4 for the data analysis. (R 10 kb)
